# Viroporins: emerging viral infection mechanisms and therapeutic targets

**DOI:** 10.1128/jvi.01038-25

**Published:** 2025-09-02

**Authors:** Yuxin Qu, Jiaqi Li, Hao Deng, Yanjin Wang, Lian-Feng Li, Bingqing Xia, Yongfeng Li, Hua-Ji Qiu, Su Li

**Affiliations:** 1 State Key Laboratory for Animal Disease Control and Prevention, Harbin Veterinary Research Institute, Chinese Academy of Agricultural Sciences 687216 , Harbin, China; 2 CAS Key Laboratory of Receptor Research, State Key Laboratory of Drug Research, Shanghai Institute of Materia Medica, Chinese Academy of Sciences, Shanghai, China; New York University Department of Microbiology, New York, New York, USA

**Keywords:** viroporins, virus life cycle, ion homeostasis, proinflammatory responses, antiviral strategies

## Abstract

Viroporins, virus-encoded small hydrophobic proteins, are involved in critical steps of viral infections by modulating ion homeostasis, disrupting host membrane integrity, and orchestrating key stages of the virus life cycle—from entry and replication to release. Beyond facilitating viral propagation, these pore-forming proteins exacerbate pathogenesis by disrupting cellular ion homeostasis and triggering proinflammatory responses through NLRP3 inflammasome activation. Their dual role in viral fitness and immunopathology positions viroporins as promising antiviral targets for dual-action therapeutics: suppressing replication while attenuating inflammation. However, structural constraints and low immunogenicity hinder vaccine development targeting conserved viroporin domains. This review summarizes recent advances in viroporin mechanisms, highlights progress in viroporin-targeted drug and vaccine design, and discusses persistent challenges. Furthermore, the emerging technologies (e.g*.*, AI-driven structural prediction and nanodelivery systems) are expected to significantly accelerate the development of next-generation viroporin-targeted therapeutics and vaccines. Taken together, this review underscores the critical roles of viroporins in viral pathogenesis, emphasizing their potential as promising targets for the prevention and control of viral diseases.

## INTRODUCTION

Viroporins are small hydrophobic transmembrane proteins encoded by viruses. They self-oligomerize to form membrane-embedded pores that facilitate ion transport, thereby regulating multiple stages of the virus life cycle and disrupting cellular metabolism and physiological processes. Viroporins were first identified in the 1970s, when increased membrane permeability was observed in picornavirus-infected cells, resulting from membrane pores formed by viral proteins (1). Viroporins exert functions at distinct stages of viral infection. Specifically, the influenza A virus (IAV) M2 protein functions as a pH-dependent proton channel to acidify the interior of the virus, triggering nucleocapsid uncoating and independently mediating membrane scission to facilitate viral budding (2, 3); mutation or deletion in the *E* gene in coronaviruses results in decreased particle production and/or aberrant virion assembly (4); the picornavirus 2B protein facilitates membrane permeability of ions and small molecules during the later stages of viral infection (5).

Recent technological advances, including predictive algorithms such as AlphaFold, a prominent example of artificial intelligence (AI), electrophysiological assays (e.g*.*, planar lipid bilayer measurements), and structural biology techniques such as cryo-electron microscopy (cryo-EM), have expanded the repertoire of identified viroporins. Notable discoveries include the Ebola virus (EBOV) delta peptide (6), the severe acute respiratory syndrome coronavirus 2 (SARS-CoV-2) E protein (7, 8), the human astrovirus XP protein (9), the potato virus Y (PVY) 6K1 protein (10), and the African swine fever virus (ASFV) proteins B117L (11) and B169L (12). However, functional redundancy and structural plasticity of viroporins pose challenges for inhibitor design, necessitating integrated mechanistic insights combined with novel technologies that may facilitate the development of next-generation viroporin-targeted countermeasures.

## CHARACTERISTICS OF VIROPORINS

The concept of viroporins stems from observations of increased membrane permeability during viral infection of host cells. In the 1970s, Carrasco obseerved that infection with picornaviruses, such as encephalomyocarditis virus (EMCV), results in increased plasma membrane permeability. This increase allows normally impermeable antiviral small-molecule inhibitors to selectively enter infected cells and specifically inhibit viral protein synthesis, a phenomenon attributed to membrane pores formed by viral proteins (1). However, it was not until 1995 that Carrasco, after identifying the proteins mediating this pore-forming activity in RNA viruses, coined the term “viroporin” (13).

Viroporins can form pores in host membranes through homo-oligomerization. These pores act as channels, increasing membrane permeability to ions and small solutes (14). Although their size can vary considerably, they are generally relatively small proteins. The large ones exhibit more than 200 amino acids (aa), including the dengue virus type 2 (DENV-2) NS2A protein (15), while the small ones are only 40–49 aa, such as the EBOV delta peptide (6).

**Fig 1:**
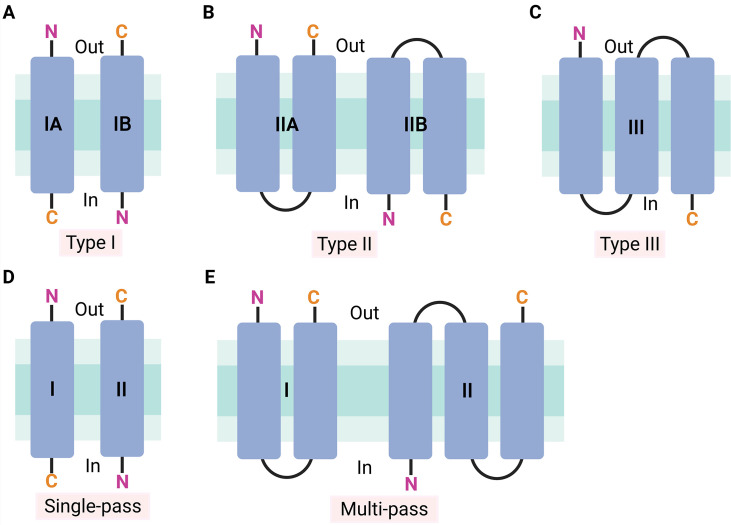
Classification schemes for viroporins based on transmembrane domain (TMD) topology. (A–C) The conventional classification of viroporins. (**A**) Type I: single TMD, subdivided by N-/C-terminus orientation. IA: luminal N-terminus, cytoplasmic C-terminus. IB: cytoplasmic N-terminus, luminal C-terminus. (**B**) Type II: two TMDs form helix-turn-helix hairpins. IIA: luminal N- and C-termini. IIB: cytoplasmic N- and C-termini. (**C**) Type III: three TMDs. (D and E) The viroporin classification scheme proposed by Devantier et al. (14). Single-pass: corresponds to the traditional classification’s type I. (**D**) Multi-pass: more than one TMD. Out: the N- or C-terminus is oriented out (lumen or extracellular). In: the N- or C-terminus is oriented in (cytosol) (**E**). The figure was generated by using the online software BioRender (https://app.biorender.com/).

Viroporins are primarily classified into types I, II, and III based on the number of their transmembrane domains (TMDs) (Fig. 1A through C). Type I contains one, type II two, and type III three TMDs, respectively (16). Types I and II are further subdivided into four subtypes according to the specific membrane orientation of their N- and C-terminal domains. Specifically, type I encompasses IA and IB, and type II includes IIA and IIB. IA-type viroporins contain a shorter N-terminal domain exposed to the luminal side, while the larger C-terminal domain is oriented toward the cytoplasmic side. In contrast, IB-type viroporins exhibit the opposite configuration. Type II viroporins form a transmembrane helix-turn-helix hairpin structure. The IIA subtype possesses luminal N- and C-termini, while the IIB subtype exhibits cytoplasmic N- and C-termini.

The functional characterization of viroporins, particularly their ion channel activity, typically employs electrophysiological techniques such as patch-clamp and planar lipid bilayers (17, 18). However, these approaches carry inherent limitations: they generally necessitate heterologous expression of candidate channels or reconstitution of isolated or synthetic peptides into artificial membranes, which may not fully recapitulate physiological cellular environments. Additionally, such methodologies exhibit significant technical variability and require rigorous validation for data interpretation. To address these challenges, McClenaghan et al. proposed that mutating key aa residues to perturb channel gating and/or ion selectivity provides a definitive criterion for confirming ion channel activity (19). Furthermore, Devantier et al. developed a checklist for identifying viroporins and established a new classification scheme (14) (Fig. 1D and E). This checklist requires both structural features (e.g*.*, TMDs, oligomeric architectures), functional proof of ion channel activity, and genetic validation. Notable cases of contentious classification include the SARS-CoV-2 open reading frame 3a (ORF3a) protein, which was initially proposed as a cation-selective viroporin based on cryo-EM structures of tetrameric assemblies, planar lipid bilayer s, and genetic validation (16, 19). However, recent studies showed that patch-clamp recordings in HEK-293 cells failed to detect currents associated with ORF3a expression, even with truncated constructs enhancing plasma membrane localization. Molecular dynamics (MD) simulations and cell swelling assays revealed ORF3a forms a water-permeable channel, with key asparagine residues (e.g*.*, N^82^, N^119^) mediating selectivity. Mutations of these key residues (e.g*.*, N82W) abolished water flux and lysosomal swelling. Therefore, the SARS-CoV-2 ORF3a protein does not qualify as a viroporin but functions as a water-permeable channel (20). This discrepancy is likely attributable to the use of heterologous expression systems such as *Xenopus* oocytes and artificial lipid membranes, which may cause abnormal protein conformation or nonspecific interactions with the host channels, generating artificial currents, without fully accounting for the physiological function of ORF3a within organelles such as lysosomes. Furthermore, early structural analyses were limited by the lack of membrane lipid environments and insufficient consideration of conformational variations within the oligomer, including differences between *cis* and *trans* protomers, potentially leading to inaccurate functional interpretation of the viroporin pore.

Viroporins exhibit significant diversity in their structure, selectivity, and gating mechanisms (Table 1). A prototypical example is IAV M2, a relatively simple viroporin that functions via a passive gating mechanism driven by pH gradients, thereby enabling cation permeability and low ion selectivity. Specifically, the His^37^ and Trp^41^ are critical for regulating proton conduction. In addition, the hepatitis C virus (HCV) p7 serves as a pH-regulated ion channel with low selectivity. Its function is more consistent with that of a passive transport pore than with highly regulated classical ion channels. The ion selectivity and gating of the HCV p7 depend on the synergistic contributions of residues such as His^17^, Val^24^, and Phe^25^, and on the architecture of its oligomeric state, which forms a flower-shaped pore from monomers, each containing two TMDs (21).

**TABLE 1:** Characteristics of the viroporins discussed in the text

Virus	Viroporin	Classification	Amino acids	Ion selectivity	Oligomeric state	PDB ID
Influenza A virus	M2	IA/SP^*d*^ type I	97	H^+^, K^+^, Na^+^	4	2KWX
Influenza B virus	BM2	IA/SP type I	109	H^+^	4	6PVR
Severe acute respiratory syndrome coronavirus 1	E	IA/SP type I	76	K^+^, Ca^2+^	5	5X29
Severe acute respiratory syndrome coronavirus 2	E	IA/SP type I	75	K^+^, Ca^2+^	2/5	7K3G
Mouse hepatitis virus	E	IA/SP type I	83	Na^+^, K^+^	–^*a*^	–
Infectious bronchitis virus	E	IA/SP type I	109	Na^+^, K^+^, Cl⁻	–	–
Poliovirus	2B	IIB/MP^*e*^ type II	97	Ca^2+^	–	–
Coxsackievirus	2B	–/MP	99	Ca^2+^, H^+^	–	–
Duck hepatitis A virus 1	2B	IA/SP type I	119	Ca^2+^	–	–
Encephalomyocarditis virus	2B	–/MP	151	Ca^2+^	–	–
Human rhinovirus	2B	–/MP	97	Ca^2+^	–	–
Foot-and-mouth disease virus	2B	IIB/MP type II	154	Ca^2+^	–	–
Ebola virus	Delta peptide	IA/SP type I	40–49	Cl⁻	–	–
Human astrovirus	XP	IA/SP type I	112	–	–	–
Potato virus Y	6K1	–/MP^*c*^	51	–	–	–
Sindbis virus	6K	–/MP^*c*^ type I	54	–	–	–
Chikungunya virus	6K	–/SP^*c*^ type I	60	–	–	–
African swine fever virus	B117L^*b*^	IA/SP type I	115	–	–	–
	B169L^*b*^	IIA/MP type I	164	–	–	–
Classical swine fever virus	p7	IIA/MP type I	69	–	–	–
Hepatitis C virus	p7	IIA/MP type I	63	Ca^2+^, K^+^, Na^+^, H^+^	6	2M6X
Human immunodeficiency virus type 1	Vpu	IA/SP type I	81	Na^+^, K^+^, Cl⁻	4/5	1PI8, 1PI7
Dengue virus type	NS2A	–/MP type I	218	–	–	–
	NS2B	–/MP type I	130	–	–	–
Mumps virus	SH^*b*^	–/–^*f*^	57	–	–	–
Parainfluenza virus 5	SH^*b*^	–/–	44	–	–	
Human respiratory syncytial virus	SH	IB/SP type II	64	–	–	2NB7, 2NB8
Human metapneumovirus	SH	IB/SP type II	179	–	–	–
Rotavirus	NSP4	–/MP	175	Ca^2+^, K^+^, Cl⁻	–	–

^*a*^ – indicates unidentified classification, ion selectivity, or viroporin structures.

^*b*^ Predicted to be a viroporin.

^*c*^ Disputed if single-pass or multi-pass.

^*d*^ SP, single-pass as shown in Fig. 1D.

^*e*^ MP, multi-pass as shown in Fig. 1E.

^*f*^ -/- indicates absence from both traditional and Devantier et al.’s (14) viroporin classification systems.

## FUNCTIONS OF VIROPORINS IN THE VIRUS AND THE HOST

The structural diversity of viroporins, including their topology, oligomeric states, and TMDs, usually determines their multifaceted functions in viral pathogenesis by regulating ion channel activity, organelle interactions, and immune responses (14, 22).

### Involvement of viroporins in the virus life cycle

Viroporins participate in various stages of the virus life cycle. Specifically, the involvement of viroporins in virus life cycle can be attributed to ion channel activity, interactions with viral or cellular proteins, or the synergistic interplay among these mechanisms.

#### Viral internalization

**Fig 2:**
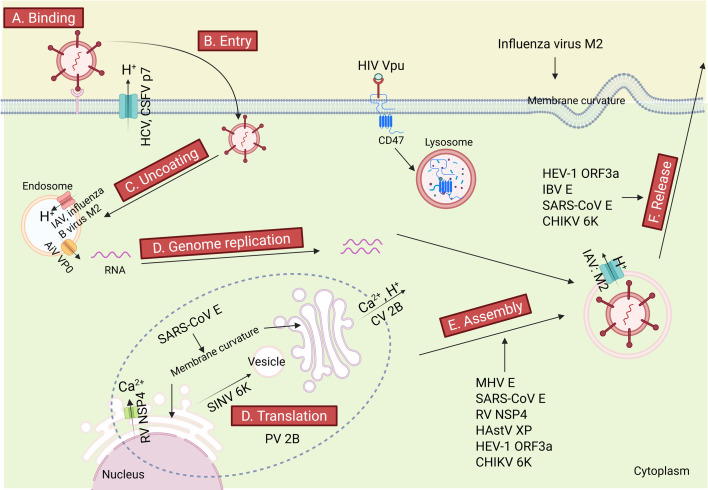
Multiple roles of viroporins in the virus life cycle. (**A and B**) Viroporins mediate viral penetration through the host plasma membrane. (**C**) Viroporins trigger conformational changes within the virion, thereby facilitating the release of viral genome into the cytoplasm. (**D**) Viroporins mediate viral replication. (**E**) Viroporins participate in the assembly of viral nucleocapsids. (**F**) Viroporins promote the release of progeny virions from infected host cells via budding or host cell lysis. HCV, hepatitis C virus; CSFV, classical swine fever virus; IAV, influenza A virus; AiV, Aichi virus; RV, rotavirus; SARS-CoV, severe acute respiratory syndrome coronavirus; SINV, Sindbis virus; PV, poliovirus; HEV-1, hepatitis E virus type 1; IBV, infectious bronchitis virus; CHIKV, Chikungunya virus; MHV, mouse hepatitis virus; HAstV, human astrovirus. The figure was generated by using the online software BioRender (https://app.biorender.com/).

The initial step of virus infection is to bind to and penetrate the host’s plasma membrane, followed by the delivery of its genome to the cytoplasm (Fig. 2A and B). Viroporins facilitate viral genome release by modulating host membrane microenvironments or directly perturbing membrane architectures (Fig. 2C). Previous studies have shown that the HCV and classical swine fever virus p7 viroporins can mediate proton efflux to establish an acidic microenvironment around virions, facilitating fusion between the viral envelope and organelle membrane (18, 23, 24). Similarly, the IAV M2 and influenza B virus BM2 viroporins both act as acid-activated proton channels that acidify the interior of the virion, thereby triggering nucleocapsid disassembly and subsequent genome release (2, 25). In addition, the Aichi virus VP0 viroporin undergoes pH-dependent oligomerization to form functional pores that facilitate viral RNA into the cytoplasm (26). Collectively, viroporins modulate membrane microenvironments across diverse virus families to facilitate membrane fusion or nucleocapsid disassembly.

#### Viral genome replication and virion assembly

Following cytoplasmic release of viral genomes, viruses orchestrate host cellular machinery through directed modulation of protein networks and organelle dynamics (Fig. 2D and E). The poliovirus 2B viroporin oligomerizes into functional membrane pores that induce structural reorganization of the endoplasmic reticulum (ER)-Golgi intermediate compartment (ERGIC), establishing replication scaffolds for viral RNA synthesis (27, 28). Furthermore, the coxsackievirus 2B viroporin forms membrane pores that mediate ion efflux (Ca^2+^/H^+^) from the ER and Golgi, thereby disrupting anterograde protein trafficking and establishing an ionic microenvironment for viral RNA replication, while disruption of the pore-forming activity of 2B abrogates the efficiency of viral RNA synthesis (29). Recently, the Sindbis virus 6K viroporin has been demonstrated to mediate ion flux that facilitates the biogenesis of cytoplasmic processing vesicles type II, and these vesicles coordinate anterograde transport of the viral envelope glycoproteins (E1/E2) from the ER to plasma membrane compartments (30). In coronaviruses, the E protein of mouse hepatitis virus (MHV) is critical for inducing membrane curvature during viral assembly, and the deletion or inactivation of the E protein results in aberrant virion morphologies (4). Moreover, the post-translationally palmitoylated MHV E protein contributes to virion assembly (31). Previously, the Chikungunya virus (CHIKV) 6K protein activity inhibited by amantadine results in the aberrant virion assembly and reduced virus infectivity (32). Elevation in cytoplasmic calcium mediated by the rotavirus (RV) NSP4 protein activates the calcium-induced signaling pathway to initiate autophagy, facilitating the trafficking of NSP4 and VP7 to “viroplasms” (the sites for viral replication) for subsequent virus assembly (33, 34). Furthermore, comparative genomic analysis and ribosome profiling revealed a conserved, functional +1 frame ORF (ORFx) overlapping the 5´ end of the capsid-coding region in astroviruses, encoding a small transmembrane protein XP. The XP protein localizes to the *trans*-Golgi network (TGN) and plasma membranes, exhibiting viroporin activity. Genetic knockout of *XP* severely attenuates virus replication, while pseudoreversion restores *XP* expression and virus proliferation (9).

#### Virus budding

Viroporins orchestrate virion release through diverse mechanisms (Fig. 2F), which can be broadly categorized into three distinct strategies: membrane curvature induction, ion flux regulation, and non-canonical pathways.

Membrane curvature induction is a common mechanism employed by enveloped viruses for budding. For instance, the M2 protein of influenza virus serves as a proton-selective ion channel. A highly conserved 17-aa amphipathic helix within its cytoplasmic tail modulates membrane curvature in a cholesterol-dependent manner and, independently, induces budding in giant unilamellar vesicles. This helix localizes to the viral budding neck, and its mutation disrupts viral membrane scission, thereby blocking particle release (3). Furthermore, upon inhibition of M2 protein function, the K58I mutant in the HA2 subunit of hemagglutinin (HA) retains its native conformation and undergoes correct transport within acidic intracellular compartments due to enhanced acid stability of HA, thereby compensating for the loss of M2 activity. This finding provides direct evidence that the M2 protein modulates the pH of compartments involved in glycoprotein trafficking through its viroporin activity, thereby protecting HA from premature inactivation caused by acidification (35).

Ion flux regulation is another critical mechanism. RV utilizes the NSP4 viroporin to trigger Ca^2+^ efflux from the ER, activating mixed lineage kinase domain-like protein-mediated necroptosis and subsequent membrane rupture to release progeny virions (36). Non-canonical pathways represent another strategy utilized by viroporins. The infectious bronchitis virus E mutants exhibit impaired viral egress, whereas revertants regain the capacity to restore ion flux and virion release, establishing the direct involvement of the E protein in the viral budding (37).

Viroporins regulate the virus life cycle by modulating host membrane microenvironments, ion homeostasis, and programmed cell death pathways. In addition to canonical membrane perturbation and ion channel functions, these proteins are also implicated in novel processes, including immune evasion.

#### Viroporin interactions with host and viral proteins for efficient proliferation

Most viroporins rely on their canonical viroporin activities to participate in viral proliferation, playing critical roles in regulating key steps of the virus life cycle. However, the precise function of several viroporins within the virus life cycle remains undefined, and some of their reported activities contributing to replication may not depend on their presumed viroporin activity. For example, the human immunodeficiency virus type 1 (HIV-1) Vpu protein is essential for efficient viral maturation and release. Deletion of the *Vpu* gene results in accumulation of viral proteins within infected T cells, promotes syncytium formation, causes retention of progeny virions at the plasma membrane, and impairs virion structural integrity (38). In addition, Vpu modulates interactions with clathrin adaptor proteins, including AP-1 and AP-2, via serine phosphorylation and tetherin binding, thereby counteracting the antiviral activities of host restriction factors. Phosphorylation-deficient mutants, such as 2/6A and EXXXLV motif mutants, display impaired tetherin antagonism, a defect reversed by fusion to clathrin-binding domains such as the HRS clathrin box (39). Furthermore, Vpu binds CD47 through its TMD and targets it for lysosomal degradation, leading to reduced CD47 surface expression on infected CD4^+^ T cells. This relieves the CD47-mediated suppression of the “don’t eat me” signal to macrophages, facilitating phagocytosis of infected cells by these immune effectors. This process represents a novel mechanism underlying early HIV-1 dissemination and macrophage reservoir establishment (40).

The cytoplasmic domains of the SARS-CoV E and M proteins specifically interact at the cytosolic face of the ERGIC, promoting the production and release of virus-like particles (VLPs). Deletion of these domains significantly impairs VLP generation. Critically, this E-M interaction facilitates membrane curvature induction during virus budding. Consistent with this role, recombinant coronaviruses lacking the E protein exhibit aberrant morphology, including constricted and elongated particles. In contrast, VLPs co-assembled from the M and E proteins display smooth, spherical contours comparable to wild-type virions (41). Reportedly, the hepatitis E virus type 1 (HEV-1) ORF3 protein interacts with host factors, including DAPK1 and histone deacetylases (HDACs). By sequestering the NF-κB component p52 (thereby implicating the non-canonical NF-κB pathway) and HDAC2, the ORF3 protein activates DAPK1. The activated DAPK1 promotes beclin-1 phosphorylation, thereby inducing beclin-1-mediated autophagy. This cascade ultimately augments HEV replication and release (42). Intriguingly, the transframe form of the CHIKV 6K protein exhibits channel activity, sharing the N-terminus with wild-type 6K (6K-WT) but possessing a distinct C-terminus that forms a structurally less complex ion channel and is incorporated into virions. Both isoforms preferentially associate with cholesterol-low ER membranes. 6K-WT requires the E2 glycoprotein for translocation to the plasma membrane to facilitate virion budding (43).

### Viroporin-induced immunopathology and antiviral interventions

**Fig 3:**
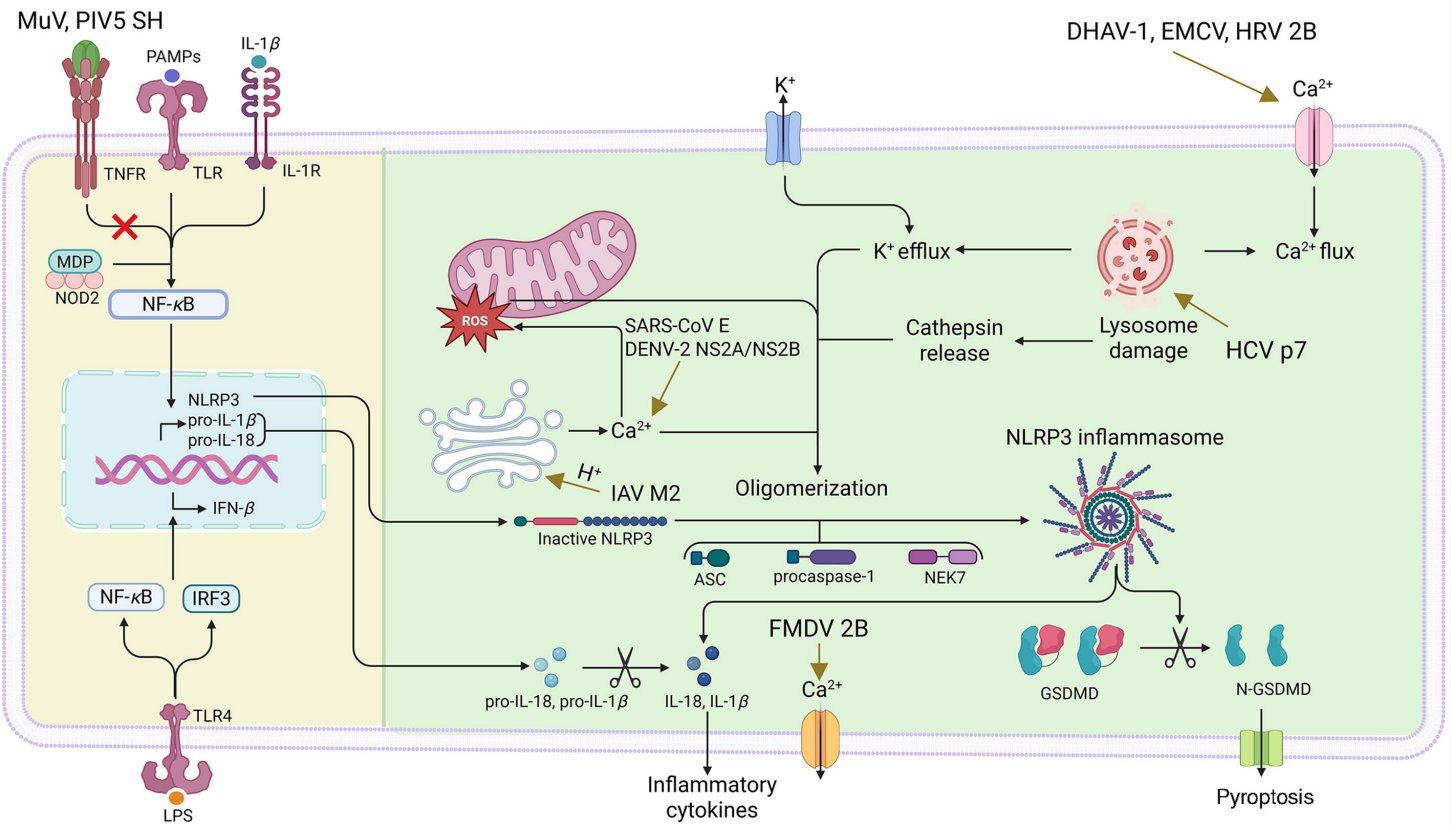
Schematic diagram of viroporin-mediated inflammatory pathways. NOD-like receptor family, pyrin domain-containing 3 (NLRP3) inflammasome is an oligomeric complex composed of the NOD-like receptor NLRP3, the apoptosis-associated speck-like protein containing a CARD (ASC), and procaspase-1. Mitochondrial damage, protein aggregation, and abnormal ion concentrations caused by viral infection can activate the NLRP3 inflammasome, leading to the secretion of interleukin (IL)-1β and IL-18 and cleavage of gasdermin D (GSDMD). Many viroporins activate the NLRP3 inflammasome by disturbing intracellular ion concentrations. Several viroporins can activate NLRP3 through mitochondrial damage-induced reactive oxygen species (ROS) production. MuV, mumps virus; PIV5, parainfluenza virus 5; DENV-2, dengue virus type 2; FMDV, foot-and-mouth disease virus; DHAV-1, duck hepatitis A virus 1; EMCV, encephalomyocarditis virus; HRV, human rhinovirus. The figure was generated by using the online software BioRender (https://app.biorender.com/).

Viroporins not only facilitate viral membrane fusion, nucleocapsid disassembly, and budding, but also contribute to pathogenesis. These proteins disrupt cellular ion homeostasis, induce inflammatory responses and apoptosis, and modulate host immune responses, ultimately leading to tissue damage and disease progression (Fig. 3). A comprehensive understanding of the pathogenesis mediated by viroporins is essential for developing targeted antiviral therapies.

NOD-like receptor thermal protein domain-associated protein 3 (NLRP3) is an intracellular sensor that activates the NLRP3 inflammasome, triggering caspase-1-dependent secretion of proinflammatory cytokines interleukin (IL)-1β and IL-18 (44). Viroporins orchestrate host proinflammatory signaling through three taxonomically classified mechanisms: (i) ion channel-mediated ion imbalance; (ii) reactive oxygen species (ROS)-dependent activation; (iii) and dual-phase immunomodulation involving both cytokine regulation and prolonged proinflammatory responses. Typically, viroporins exhibit cell type-specific plasticity and host environment-dependent functional divergence, warranting case-specific analysis based on spatiotemporal context (45).

#### Ion imbalance-driven inflammation

Viroporins activate the NLRP3 inflammasome by inducing ionic imbalance or osmotic stress through their channel activity—a mechanism shared by multiple RNA viruses, including influenza viruses and coronaviruses. For instance, the 2B protein of duck hepatitis A virus 1 elevates intracellular Ca^2+^ levels, thereby activating the NF-κB and NLRP3 signaling pathways, facilitating IL-1β production (46). Similarly, the ion channel activity of the SARS-CoV E protein disrupts cellular ion homeostasis, primarily by mediating Ca^2+^ release from the ERGIC and Golgi apparatus into the cytosol. This Ca^2+^ efflux elevates cytosolic Ca^2+^ levels, thereby triggering NLRP3 inflammasome activation (47, 48). Furthermore, the EMCV and human rhinovirus 2B proteins activate the NLRP3 inflammasome via intracellular Ca^2+^ overload, which is inhibited by the calcium chelator BAPTA-AM [1,2-*bis*-(ortho-aminophenoxy)ethane-N,N,N',N'-tetraacetic acid tetrakis (acetoxymethyl) ester] or the channel blocker verapamil (49). Additionally, the foot-and-mouth disease virus 2B protein activates the NLRP3 inflammasome and releases IL-1β through Ca^2+^ efflux. The 2B protein depends on aa 140–145 for ion channel formation. A substitution mutation of these residues that alters hydrophobicity was shown to impair the NLRP3 inflammasome activation triggered by the 2B protein (50). Recently, the viroporin activity of RV NSP4 has been shown to trigger intercellular calcium waves that contribute to viral pathogenesis (51). Collectively, viroporin-induced ionic concentration changes, especially Ca^2+^, serve as the “second signal” required for NLRP3 inflammasome activation.

#### ROS-dependent inflammasome activation

ROS serves as pivotal signaling molecules in viroporin-induced proinflammatory responses. The SARS-CoV-2 ORF3a and E (a viroporin) proteins both activate the NLRP3 inflammasome via the mitochondrial permeability transition pore. The two proteins synergistically increase cytoplasmic and mitochondrial Ca^2+^ concentrations, promoting the tricarboxylic acid cycle to generate excessive NADH, which in turn leads to the production of mitochondrial ROS (52). Similarly, the DENV-2 NS2A/NS2B proteins promote ROS accumulation through Ca^2+^ overload-induced mitochondrial membrane depolarization, leading to NLRP3 inflammasome activation (53). Notably, the IAV M2 protein modulates the pH environment of TGN, indirectly enhancing ROS accumulation and providing a physical platform for NLRP3 inflammasome oligomerization (54). Additionally, the HCV p7 protein disrupts lysosomal acidification, inducing pH imbalance and ROS release and activating NLRP3 (55). Taken together, viroporins disrupt mitochondrial functions or organelle homeostasis, thereby generating ROS, which acts as a trigger for inflammasome activation.

#### Immunomodulation strategies

Viroporins can prolong proinflammatory responses by suppressing host anti-inflammatory pathways or immune surveillance. It has been shown that the HIV-1 Vpu protein induces chronic inflammation through ubiquitin-mediated degradation of CD4, suppression of IL-21 secretion, and impairment of dendritic cell maturation, thereby attenuating adaptive immunity (56). The SH protein of paramyxoviruses (e.g., mumps virus, parainfluenza virus 5) has been demonstrated to bind to tumor necrosis factor receptor 1 (TNFR1) for inhibition of the NF-κB signaling, thereby reducing TNF-α secretion and attenuating the apoptosis-mediated inflammatory cascades (57).

#### Cell-type and host-environment dependency in immunomodulation

The inflammatory regulatory functions of certain viroporins exhibit marked cell-type and host-environment specificity. The SH protein of human respiratory syncytial virus (HRSV) functions as a viroporin that activates the NLRP3 inflammasome and promotes IL-1β secretion in lung epithelial cells, yet paradoxically suppresses IL-1β production in immune cells. While SH typically inhibits TNF secretion *in vitro*, the *SH* gene-knockout mice infected with HRSV paradoxically show elevated pulmonary IL-6 and CXCL1 levels, demonstrating a microenvironment-dependent immunomodulation (57). The human metapneumovirus SH protein was shown to activate the NLRP3 inflammasome, thereby promoting IL-1β maturation through induction of caspase-1 cleavage and exacerbating inflammation and disease severity in infected mice. Conversely, deletion of the SH protein or inhibition of NLRP3 attenuates weight loss, mortality, and inflammatory responses (58).

Overall, viroporin-mediated inflammatory activation exhibits conserved mechanisms (e.g*.*, ion channel activity and ROS-dependent pathway) alongside virus-specific strategies (e.g*.*, organelle-targeted manipulation) and host microenvironment-dependent regulation (e.g*.*, dual regulatory functions of the HRSV SH protein).

## VIROPORIN-TARGETED ANTIVIRAL STRATEGIES

### Development of antivirals targeting viroporins

**Fig 4:**
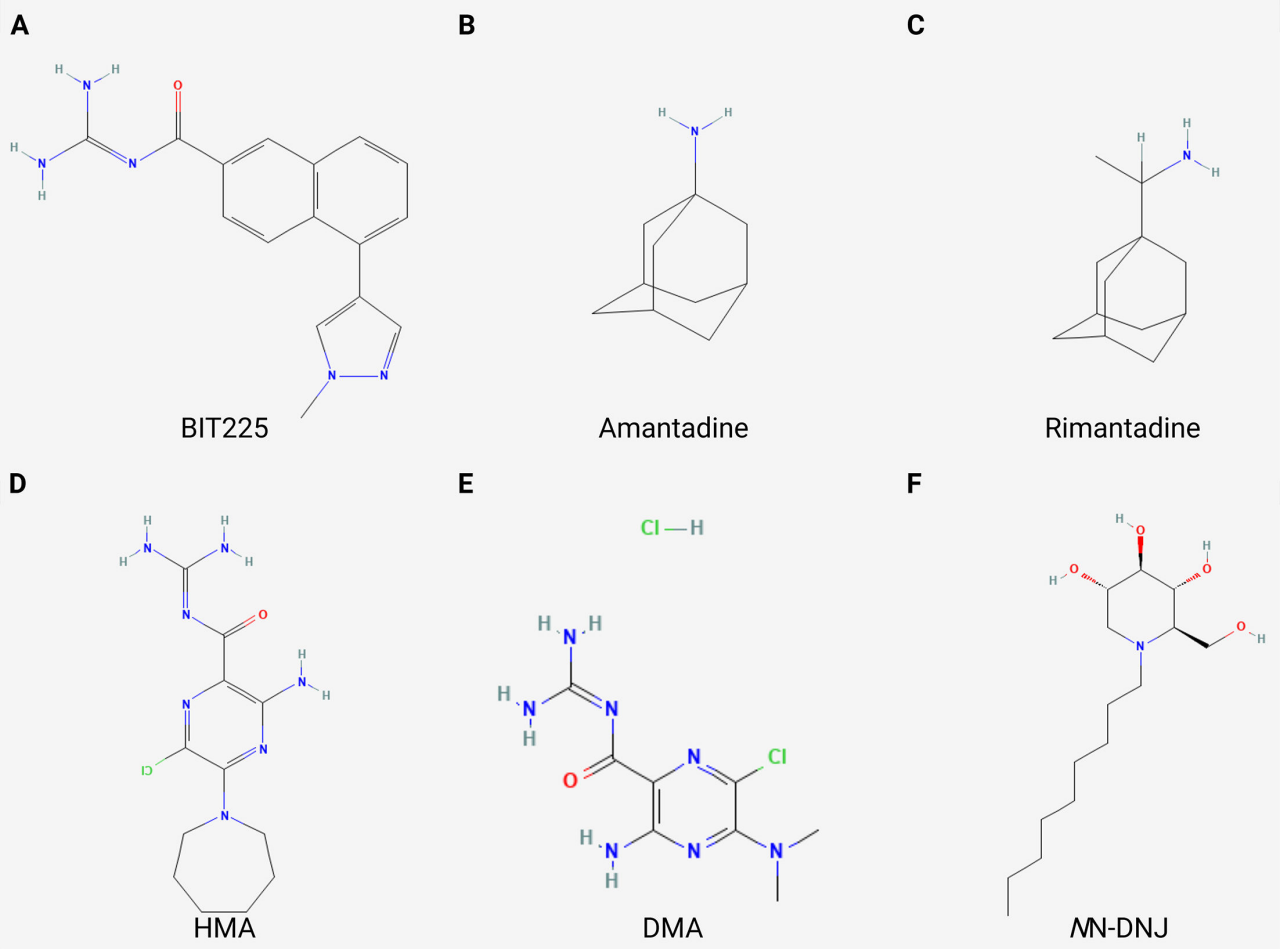
Chemical structures of various viroporin inhibitors. **(A)** BIT225. PubChem Identifier: CID 12004418. URL: https://pubchem.ncbi.nlm.nih.gov/compound/12004418#section=2D-Structure. (**B**) Amantadine. PubChem Identifier: CID 2130. URL: https://pubchem.ncbi.nlm.nih.gov/compound/2130#section=2D-Structure.** (C)** Rimantadine. PubChem Identifier: CID 5071. URL: https://pubchem.ncbi.nlm.nih.gov/compound/5071#section=2D-Structure. (**D**) Hexamethylene amiloride (HMA). PubChem Identifier: CID 1794. URL: https://pubchem.ncbi.nlm.nih.gov/compound/1794#section=2D-Structure.** (E)** Dimethyl amiloride (DMA). PubChem Identifier: CID 11957442. URL: https://pubchem.ncbi.nlm.nih.gov/compound/11957442#section=2D-Structure. (**F**) *N*-nonyl deoxynojirimycin (*N*N-DNJ) . PubChem Identifier: CID 501640. URL: https://pubchem.ncbi.nlm.nih.gov/compound/501640#section=2D-Structure. The merged figure was generated using the online software BioRender (https://app.biorender.com/).

Current antiviral drug development targeting viroporins focuses primarily on small-molecule inhibitors that directly antagonize ion channel activity or disrupt channel formation (Fig. 4). The M2 proton channel blockers (e.g*.*, amantadine and its derivatives) suppress IAV replication by inhibiting proton conductance, thereby interfering with viral uncoating, assembly, and budding (59, 60). In addition to influenza viruses, inhibitors targeting viroporins of other viruses have also demonstrated therapeutic potential. For example, the SARS-CoV-2 E channel inhibitor BE-33 significantly reduced the virus loading and weakened inflammation in the lung when the drug was administered after infection (7). BIT225, a small-molecule inhibitor, selectively targets the ion channel activity of the HIV-1 Vpu protein by blocking its oligomerization, thereby suppressing virion release, particularly from myeloid lineage cells, such as macrophages. A phase II clinical trial revealed that BIT225 exhibited antiviral activity and immunomodulatory effects with a manageable safety profile. However, its risk of drug resistance and long-term toxicological profile require further evaluation in large-scale clinical trials (56, 61). Notably, BIT225 also inhibits HCV p7 ion channel activity to suppress viral genome replication (62). Additionally, hexamethylene amiloride and dimethyl amiloride suppress HIV-1 replication in monocyte-derived macrophages, demonstrating significant antiviral efficacy through blockade of Vpu-mediated ion channel activity (63). Reportedly, the iminosugar derivative *N*-nonyl deoxynojirimycin (*N*N-DNJ) exerts its antiviral effect by incorporating into the HCV p7 monomer, thereby inhibiting ion channel oligomerization. Mechanistically, the disruption of quaternary structure assembly by *N*N-DNJ impedes functional channel formation and subsequent suppression of virion release (64).

Currently, the only clinically approved viroporin-targeting therapeutics is adamantane derivatives (e.g*.*, amantadine) directed against IAV M2 proton channels. However, due to widespread resistance, their routine clinical use has been discontinued (59, 60). The SARS-CoV-2 pandemic, which began in 2019, has reignited scientific interest in amantadine as a potential repurposed therapeutic agent against viral infections. A randomized, double-blind, placebo-controlled trial in non-hospitalized adults with COVID-19 found that amantadine (100 mg twice daily for 5 days) showed no significant benefit over placebo in clinical improvement, hospitalization rates, or serious adverse events (65). Novel viroporin inhibitors targeting HIV remain confined to preclinical development, yet already emerge as promising candidates for next-generation antiviral therapies (56). As emerging and attractive antiviral targets, viroporin inhibitors offer a promising therapeutic strategy with dual mechanisms: direct suppression of viral replication and mitigation of inflammatory damage to the host mediated by viroporins. This dual functionality stems from the intrinsic role of viroporins as bridges between viral propagation and host immunopathology, thereby enabling a single inhibitor to concurrently target both processes.

### Development of vaccines

Small-molecule inhibitors exhibit significant therapeutic potential but encounter notable challenges in clinical application. The rapid emergence of drug-resistant viral strains, as exemplified by adamantane-resistant influenza variants, highlights the urgent requirement for complementary antiviral strategies. This challenge has spurred growing interest in developing vaccines targeting conserved viroporin epitopes, potentially providing a viable preventive option.

Viroporins are crucial for viral replication and contribute to viral pathogenesis. Therefore, viruses lacking these proteins can be used as attenuated live vaccines to combat viral infections. Moreover, many viroporins exhibit highly conserved aa sequences across various virus strains, indicating the potential immunogenicity. The conservation enables vaccines based on viroporins to offer cross-protection against multiple viral strains. However, current studies on the development of vaccines targeting viroporins remain limited, with a primary focus on the IAV M2 protein and the SARS-CoV E protein.

The influenza virus mutant lacking the *M2* gene showed reduced viral replication in cell cultures and attenuated virulence in a mouse model. Mice intranasally inoculated with the *M2*-deleted elicited protective immune responses and survived lethal wild-type virus challenge, indicating its potential as a candidate live attenuated vaccine (66).

Additionally, the full-length M2 protein was successfully reconstituted by using nanodiscs, mimicking its natural transmembrane structure in the viral envelope. Synthetic cytosine-phosphate-guanine oligodeoxynucleotides, an adjuvant that enhances immune responses through Toll-like receptor 9 activation, were incorporated into nanostructures. Immunization via intranasal and intramuscular routes led to a marked reduction in viral load in both the upper and lower respiratory tracts of pigs and completely eliminated pneumonia in 89% of vaccinated animals, demonstrating the efficacy of this vaccine candidate (67).

The aa sequences of the M2 protein extracellular domain (M2e) are highly conserved, and the anti-M2e sera can inhibit influenza virus replication. However, due to its short length (23 aa), M2e displays weak immunogenicity. Notably, a vaccine targeting the *M2e* gene and utilizing the hepatitis B core protein as a carrier was developed to construct the M2e-HB VLPs vaccine, and the vaccine elicited a robust antibody response in mice (68, 69). In addition, researchers developed an M2e-based VLPs delivered by human papillomavirus. When combined with an aluminum salt adjuvant, it induced a robust immune response in mice (70). Recently, 2020/2021 seasonal influenza virus H3N2 VLPs expressing either the M2e5x antigen (H3N2-M2e5x VLPs) or the N2 antigen (H3N2-N2 VLPs) have been developed (71). Interestingly, both VLPs induced cross-protective immunity against heterologous influenza viruses. Notably, the H3N2-M2e5x VLPs significantly reduced lung viral titers following challenge with H1N1, H3N2, or H5N1 strains, indicating that VLPs incorporating the M2e5x antigen represent a promising strategy for universal influenza vaccine development. Additionally, M2 has high hydrophobicity and is insoluble in conventional vaccine solvents after purification. Therefore, improving the expression levels and solubility of M2 protein remains a pressing challenge.

Reportedly, the recombinant SARS-CoV with the *E* gene knockout, derived from the Urbani strain, provided effective protection in young mice against the lethal challenge with a mouse-adapted SARS-CoV strain (MA15) (72). To enhance vaccine efficacy, researchers have developed a second candidate vaccine with the *E* gene knockout in the MA15 background (rMA15-ΔE). This vaccine provided complete protection in 6-week-old, 12-month-old, and 18-month-old mice from lethal virus challenge(73). Additionally, substitution of the N-terminus of the SARS-CoV E protein or deletion of the internal C-terminal region attenuated virulence. In particular, when compared to the wild-type virus, the mutant viruses induced minimal lung injury in BALB/c mice, showed reduced neutrophil infiltration, and had higher numbers of CD4^+^ and CD8^+^ T cells (74).

Although preclinical studies have shown promising efficacy, vaccine development targeting conserved viroporin domains encounters distinct translational challenges. The intrinsic structural properties of viroporins, particularly their hydrophobic TMDs and pH-dependent conformational changes, pose significant obstacles to immunogen design and recombinant expression. These technical hurdles have prompted the adoption of innovative computational approaches, including structure-guided antigen design, coupled with advanced delivery platforms to enhance immunogenicity.

### Emerging technologies in the development of antiviral strategies

Recent technological advances have significantly enhanced the efficiency of targeted new drug research and development. Importantly, cryo-EM provides static high-resolution frameworks, whereas MD simulations reveal dynamic behaviors. Their integration enables the identification of comprehensive characterization of viroporin, including ion channel gating states (open/closed conformations). Specifically, cryo-EM has resolved the three-dimensional structure of ORF3a protein embedded in lipid nanodiscs, revealing a tetrameric assembly with a central polar cavity postulated to represent a putative ion-conducting pathway (75). In addition, cryo-EM has resolved the homodimeric architecture of SARS-CoV-2 M protein in lipid nanodiscs, revealing a cytoplasmic domain with a pronounced positively charged surface (76). MD simulations demonstrate that M protein maintains high rigidity within lipid bilayers, which is consistent with its function as a structural scaffold that ensures viral envelope integrity. The conserved characteristics of the M protein and its electropositive surface offer a structural blueprint for developing broad-spectrum antivirals targeting assembly processes (e.g*.*, disrupting M-N or M-RNA interactions).

Inspiringly, the emergence of AI technology has introduced significant breakthroughs in this field. Combining structural insights from cryo-EM with AI-driven predictions accelerates viroporin-targeted drug discovery. It has been shown that AlphaFold2 was employed to predict the structure of the 6K1 protein. The monomer was found to adopt a helix-turn-helix fold with a hairpin-like topology and short N- and C-termini. Among the predicted tetrameric to heptameric assemblies, the 6K1 monomers formed an α-helical bundle, with the pentamers exhibiting the highest predicted confidence score. Further analysis showed that the pentameric 6K1 harbored a funnel-shaped hydrophobic tunnel at its center, with a minimum radius of around 2.0 Å, compatible with the permeation of most cations. These structural features suggest that 6K1 functions as a viroporin (10). AI leverages advanced computational algorithms and machine learning models to accelerate structural predictions of viroporins, enhancing predictive accuracy while significantly reducing temporal costs and enabling rapid identification of potential therapeutic targets (77). AI-driven virtual screening optimizes prediction of novel protein-ligand interactions, enhancing drug discovery efficiency through systematic identification of viroporin-targeting compounds (78, 79).

In addition to target identification, novel drug delivery systems also enhance the therapeutic efficacy of drugs. For instance, nanoparticle-based drug delivery systems improve the solubility and stability of drugs, facilitating their release at the target site and enhancing bioavailability. By modifying nanoparticle carriers to be target-specific, drugs can be precisely delivered to the virus-infected cells, thereby minimizing their distribution to non-target organs and reducing damage to normal tissues. Moreover, nanoparticles have extended-release characteristics, facilitating sustained and controlled drug release within the host, thereby prolonging the duration of therapeutic effect and reducing the frequency of drug administration (80–82). A “photoswitchable mechanism” can be integrated into drug molecules, where light exposure induces structural changes, thereby modifying their pharmacological properties. By employing a photoswitchable mechanism to control drug activity, precise spatiotemporal activation can be achieved, enabling targeted therapy *via* spatiotemporally controlled drug delivery, effectively minimizing systemic side effects (83, 84).

## CONCLUSION AND OUTLOOK

Research to date has defined the structures and functional modes of various viroporins. However, a comprehensive understanding of their ion channel biophysics such as selectivity and gating, their involvement in cellular regulatory networks, and dynamic spatiotemporal roles in virion assembly and release is still incomplete. Elucidating how viroporins exploit host signaling pathways to enable efficient viral replication remains an important research focus. In addition, very few viroporin-targeting inhibitors have been clinically approved. Notably, adamantane derivatives, which target the IAV M2 protein, were approved for clinical use but are no longer recommended due to widespread drug resistance. BIT225 remains in clinical trials. These inhibitors are limited by a narrow antiviral spectrum, low efficacy, and the emergence of resistance. Future efforts should prioritize the development of highly specific inhibitors featuring optimized structures, improved bioavailability, and resistance-mitigation potential. One promising improvement strategy may entail the development of antiviral cocktail therapies that simultaneously incorporate inhibitors of viral protease, polymerase, and viroporin. This combinatorial approach has the potential to enhance antiviral efficacy by targeting multiple stages of the virus life cycle. For example, BIT225 shows safety and modulates immune/inflammatory markers when added to standard antiretroviral therapy (ART) in treatment-naïve individuals. Although viral load suppression mirrors ART alone, BIT225 reduces the macrophage inflammation marker sCD163 and increases activated CD4^+^/CD8^+^ T cells and NK cells. This targets Vpu-mediated immune evasion and viral release from myeloid cells, mechanisms unaddressed by conventional ART. By inhibiting the Vpu ion channel and restriction factor antagonism, such cocktails may enhance viral clearance and immune restoration, complementing ART’s inhibition of viral replication (56). For vaccines targeting viroporin antigens, such as the M2e of IAV, challenges include enhancing antigen immunogenicity, expression levels, and scalable production. Innovations in adjuvants, delivery systems, and gene editing or synthetic biology tools, such as those enabling optimized expression vectors, could accelerate vaccine development and clinical testing. Moving beyond therapeutics and vaccines, the development of precise intervention tools, such as dynamic structural conformation capture techniques based on cryo-EM and light-responsive nanoparticle delivery systems, should be developed to improve targeting specificity and spatiotemporal precision. Furthermore, interdisciplinary collaboration, including synthetic biology, AI, and computational chemistry, will accelerate the discovery and optimization of pharmacologically actionable targets. Research of viroporins shows great promise for significant breakthroughs, providing innovative solutions to tackle the challenges posed by viral diseases.
